# Mitigating risks of malaria and other vector-borne diseases in the new capital city of Indonesia

**DOI:** 10.1038/s41467-024-54891-x

**Published:** 2024-12-05

**Authors:** Henry Surendra, Bimandra A. Djaafara, Helen D. Prameswari, Dedy Supriyanto, Ponco Waluyo, Setyo B. Basuki, Herdiana Herdiana, Ermi Ndoen, Siswanto Siswanto, Inke ND Lubis, Xiaoyue Liu, Swapnil Mishra, Kimberly M. Fornace, Iqbal RF Elyazar

**Affiliations:** 1 Public Health Program, Monash University Indonesia, Tangerang, Indonesia; 2 Oxford University Clinical Research Unit, Faculty of Medicine, Universitas Indonesia, Jakarta, Indonesia; 3 Saw Swee Hock School of Public Health, National University of Singapore, Singapore, Singapore; 4 National Malaria Programme, Ministry of Health of Indonesia, Jakarta, Indonesia; 5 District Health Office of Penajam Paser Utara, Penajam Paser Utara, Indonesia; 6 Province Health Office of East Kalimantan, Samarinda, Indonesia; 7 World Health Organization, Jakarta, Indonesia; 8 United Nations Children’s Fund, Jakarta, Indonesia; 9 Faculty of Public Health, Universitas Mulawarman, Samarinda, Indonesia; 10 Faculty of Medicine, Universitas Sumatera Utara, Medan, Indonesia

**Keywords:** Malaria, Environmental impact

## Abstract

Indonesia is developing a new capital city, Ibu Koata Nusantara, which is located in a malaria and biodiversity hotspot. In this Comment, the authors outline some of the potential infectious disease-related risks of the new project – primarily malaria and other vector-borne diseases.

## Teaser

The development of Ibu Kota Nusantara, the new capital city of Indonesia, is one of the largest infrastructure projects in Southeast Asia. Targeted surveillance and adaptive control measures are needed to mitigate the risks of malaria and other infectious diseases.

**Fig. 1:**
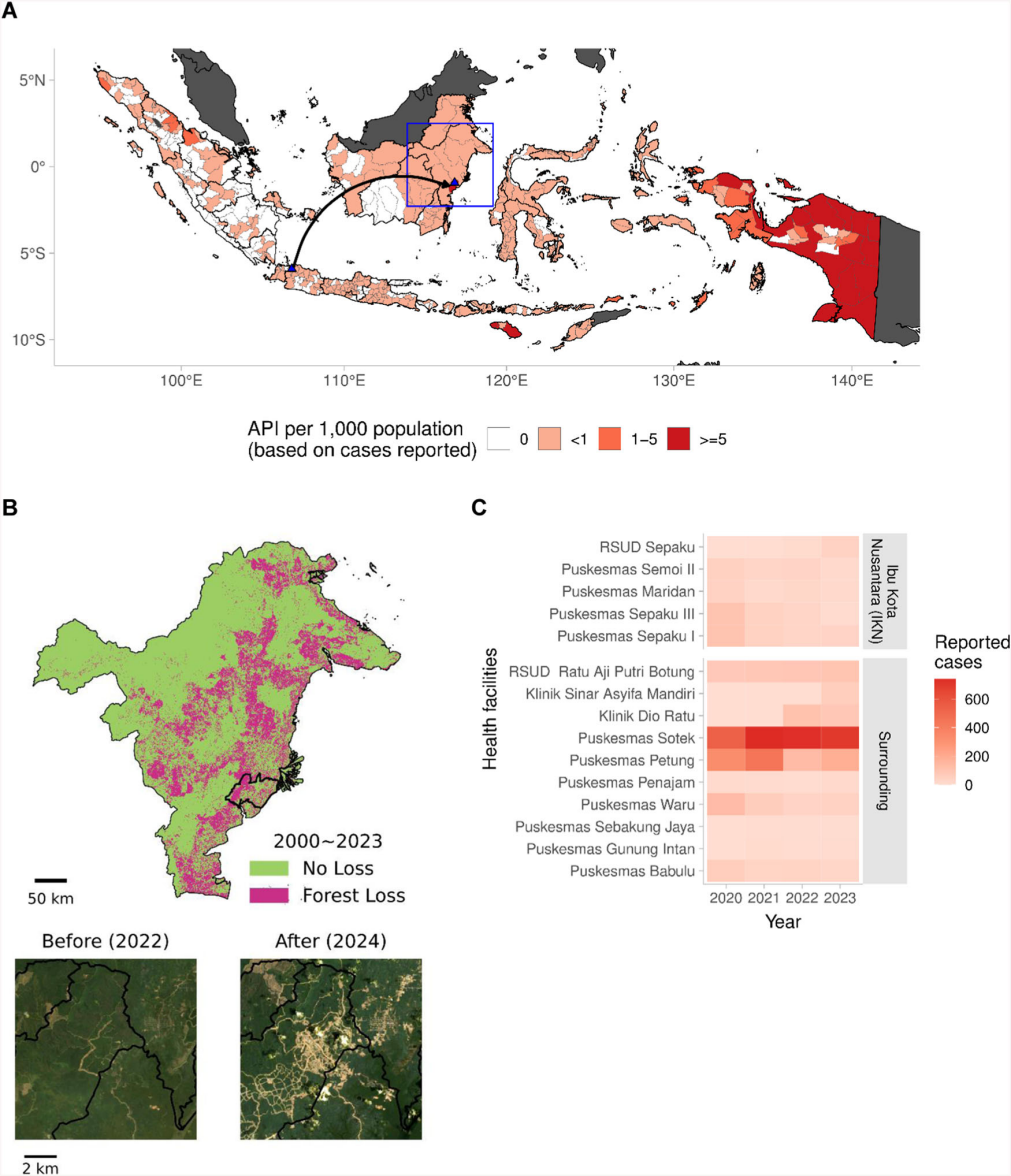
Implications of relocation of Indonesian capital city from Jakarta to Kalimantan. Maps showing the relocation of Indonesia’s capital city form malaria-free areas in Jakarta to a new city located in a malaria hotspot in East Kalimantan (**A**), forest loss, and landscape of the Indonesia’s new capital city before development in 2022 and on after development in 2024 (**B**), and number of reported malaria cases in health facility located in Ibu Kota Nusantara and surrounding areas 2022–2024* (**C**). Data sources: Malaria data were collected from the Indonesian Malaria Surveillance and Information System. Shape file was from https://data.humdata.org/dataset/cod-ab-idn. Landsat-8 image courtesy of the U.S. Geological Survey (https://www.usgs.gov/centers/eros/data-citation). Forest Coverage: Hansen MC, Potapov PV, Moore R, et al. 2013. High-Resolution Global Maps of 21st-Century Forest Cover Change. Science 342 (15 November): 850-53. https://glad.earthengine.app/view/global-forest-change. *Malaria data in (**C**) were based on report as per May 2024.

Large-scale infrastructure projects disrupt existing ecosystems and can create new opportunities for infectious disease transmission. One of the most ambitious infrastructure projects in Southeast Asia is the relocation of the capital city of Indonesia from malaria-free areas in Jakarta to Ibu Kota Nusantara (IKN), a new city located in a malaria and biodiversity hotspot in East Kalimantan (Fig. 1A). Despite goals to create a sustainable capital city, the large-scale disruption and clearing of forests associated with the development of planned urban centres, and the expansion of roads and associated industries have the potential for extensive environmental degradation both within the new development and surrounding areas (Fig. 1B)^1^. Further, the commitment to create a climate-friendly city by maintaining at least 75% green space within IKN presents new challenges and opportunities for urban design to support public health.

## Emerging risk of malaria and other vector-borne diseases in an era of environmental change

Land use changes, such as deforestation, urbanisation and expanding highway networks, are one of the main drivers of vector-borne disease transmission. These changes can create new vector habitats, alter the behaviour, distribution and species composition of mosquitoes and wildlife reservoirs and lead to the movement of susceptible or infected human populations into new areas^2^. Across Southeast Asia, malaria transmission primarily occurs in forested regions and is associated with occupational activities^3^. North of IKN, in Sabah, Malaysia, extensive deforestation is strongly associated with the emergence of the zoonotic malaria *Plasmodium knowlesi*^4^. Carried by non-human primates with human cases driven predominantly by zoonotic spillover, *P. knowlesi* poses a major barrier to malaria elimination across Southeast Asia and has no demonstrated effective control measures^5^. The public health threat caused by *P. knowlesi* appears to be growing with numbers of *P. knowlesi* cases in humans increasing across Southeast Asia, with the highest risks in populations who live near degraded forested areas^6^.

Southeast Asia has made substantial progress in malaria control and aims to achieve malaria elimination by 2030. However, malaria remains a major public health problem in many areas of Indonesia^7^, with one of the highest malaria burdens in the region and an estimated 1,156,000 cases in 2022^8^. All five species of *Plasmodium* have been reported in Indonesia including the newly emerging *P. knowlesi*^6^. Despite national reductions in malaria incidence, many provinces remain malaria-endemic, leaving significant proportions of the population at risk^7^. Existing diagnostics and clinic-based surveillance methods often miss a large proportion of infected individuals, leading to resurgence of transmission^9^. These challenges are amplified by difficulties capturing infections among high-risk mobile and migrant populations such as migrant workers, forest loggers and indigenous populations. There is an urgent need to identify high-risk environments to both target surveillance and control measures and ensure future development minimizes disease risks.

Despite the significant progress in controlling malaria in East Kalimantan, incidence of malaria remains highly spatially heterogeneous, with most areas of the new capital city remaining at high-risk of malaria transmission (Fig. 1C). Based on local case investigations, malaria infections in East Kalimantan are associated with overnight forest-related activities such as logging and watershed rehabilitation and imported infections among migrant workers coming from malaria endemic settings; both factors are likely to increase with the development of IKN^10^. Additionally, long term establishment of IKN will have profound effects on travel and trade networks around the region and result in influxes of civil servants and other populations without previous malaria exposure^10^. These expanding highway and trade networks are also strongly linked with encroachment into wildlife habitats, changes in ecological community structures with higher numbers of zoonotic reservoirs and increasing spillover of zoonotic pathogens^11^. Recently, in May 2024, one human PCR-confirmed *P. knowlesi* case was detected in the IKN surrounding areas and there is a need for further surveillance of *P. knowlesi* and other emerging zoonotic pathogens.

As in many other countries in Southeast Asia, Indonesia also faces serious challenges controlling other vector-borne and potentially zoonotic diseases, such as dengue and chikungunya, with increased burdens strongly associated with urbanisation and changing climates^12^. Many arbovirus vectors, such as *Aedes aegypti*, are highly adapted to urban environments and infrastructure development may expand potential breeding sites. However, there is limited access to quality diagnostics and lack of surveillance for dengue, chikungunya and other arboviruses, such as Japanese encephalitis virus^13,14^. East Kalimantan has the fifth highest dengue incidence rate (147 per 100,000 population) in Indonesia^15^, with very little evidence is available for other vector-borne diseases. While development may increase suitable habitats for vectors or wildlife hosts associated with human-modified landscapes, these changes also impact wider ecological community structures and biodiversity, with potentially complex impacts on disease emergence and transmission. These shared challenges offer a unique opportunity for disease control programmes to integrate surveillance for malaria and other vector-borne diseases as well as develop novel surveillance approaches to assess emerging disease risks.

## Targeted disease surveillance and control across large-scale infrastructure developments

With advanced planning of large-scale developments, there is an opportunity to proactively develop infectious disease surveillance and control measures in response to environmental changes. Within Indonesia, a Malaria Free Task Force was established by the IKN authority on 31 May 2024 to enhance surveillance and target control measures for high-risk populations. This includes malaria screening for new construction workers developing the IKN, vector control measures such as distribution and monitoring of Long-Lasting Insecticidal Nets (LLINs) and insecticide residual spraying (IRS) in labourers’ dormitories and larval monitoring and management across IKN. In the surrounding areas from IKN, measures include LLIN for pregnant women and mobile and migrant populations such as migrant workers, forest loggers, rubber tappers, watershed rehabilitation workers, and other populations working and spending nights in the forest. Surveillance and response will be strengthened by ensuring each new case is investigated promptly, conducting screening among migrant workers entering and leaving, biweekly mass fever screening, and reactivating the forest malaria stations responsible for malaria testing and treatment for people working in the forest. Within IKN, regular malaria testing will be conducted for project staff. However, screening outside the IKN project presents difficulties, though, as some of the personnel outside the project are undocumented and environmental changes extend across Borneo. Starting in September 2023, the IKN authority has implemented a pilot measure that include distribution of RDT, repellent, LLIN, and chemoprophylaxis for undocumented populations, a measure that will be implemented to all East Kalimantan areas in 2025. Ultimately, there is a need for cross-border, proactive surveillance of the impacts of environmental changes on disease risks.

Additionally, based on our field observations, construction activities can lead to creation of new vector habitats, such as standing water from project operations related to IKN. These puddles are caused by a variety of things, including heavy machinery used to create water basins, falling timber, vehicles on deserted train tracks, illegal labourers excavating land, and more. Numerous waterbody types have been shown to harbour *Anopheles* mosquito larvae.

## Research and policy priorities

Within IKN, the Malaria Free Task Force exemplifies how a disease control programme can respond to new disease risks from large-scale environmental changes and the movement of people and workers into a disease-endemic region. However, there is a need for further research to assess the current dynamics and potential risks of malaria and emerging vector-borne diseases (particularly *P. knowlesi*), and to formulate how to best mitigate the risks in the context of massive landscape changes, vector and wildlife distribution, and human determinants (mobility, immunity, and healthcare access). With the increasing availability of satellite-based remote sensing data, climatic forecasts and artificial intelligence tools to understand large and complex datasets, there is increased potential to monitor environmental changes in near real-time and better target surveillance and control activities. Investing in collecting high quality epidemiological datasets and surveillance of vector and wildlife populations alongside environmental data offers the opportunity to not only understand these changes, but also refine urban development plans to mitigate emerging risks.

Large-scale infrastructure projects need to account for a wide range of priorities, from economic development to conservation, climate mitigation and health. Economic development and expanding infrastructure will increase access to healthcare and socioeconomic status of IKN inhabitants and surrounding populations, with expected benefits for public health. However, there is a need to mitigate potential negative impacts and examine how sustainable development can support varied conservation, economic and health goals in the wider context of global environmental change. IKN is not the first large-scale development globally; other countries have established new capital cities such as Naypyidaw in Myanmar and Brasilia in Brazil. As climate change effects, such as flooding, are main drivers of the movement of the capital city from Jakarta to Kalimantan, other countries will likely face similar challenges and need to develop new cities. The development of IKN in a malaria hotspot and corresponding commitments to proactive malaria surveillance and control measures offer unique lessons on how disease control programmes can approach these large-scale infrastructure projects. However, further research is required to develop surveillance systems and policies to mitigate risks of multiple vector-borne and zoonotic diseases from both known and unknown pathogens in areas experiencing extensive environmental change. With the impacts of IKN extending across Borneo, this will require cross-border collaboration and multidisciplinary approaches to monitor health, social and ecological changes.
